# Biomarkers in neonatal encephalopathy: the role of high-mobility group box 1 in prognosis and potential therapy

**DOI:** 10.1038/s41390-025-04309-1

**Published:** 2025-09-10

**Authors:** Eleanor J. Molloy

**Affiliations:** 1 Discipline of Paediatrics, Trinity College Dublin, the University of Dublin, Dublin, Ireland; 2 Trinity Translational Medicine Institute (TTMI), St James Hospital & Trinity Research in Childhood Centre (TRiCC), Dublin, Ireland; 3 Neurodisability, Children’s Health Ireland (CHI) at Tallaght, Dublin, Ireland; 4 Neonatology, CHI at Crumlin, Dublin, Ireland; 5 Paediatrics, Coombe Hospital, Dublin, Ireland

## Need for validated standardised biomarkers in neonatal encephalopathy

Neonatal encephalopathy (NE) affects 1.15 m babies per annum globally and is an important cause of neonatal death and disability such as Cerebral Palsy.^1^ Standardised blood biomarkers of severity, prognosis and response to therapy are lacking although multiple biomarkers have been described and validated. Systemic inflammation is associated with the severity of NE as well as the neuroimaging and developmental outcomes.^2,3^

## Inflammatory biomarkers in NE

The systemic inflammatory response with both pro and anti-inflammatory cytokines responses is only mildly affected by therapeutic hypothermia.^2–8^ Further understanding of the pathophysiology, phases of injury, and therapeutic windows for present and future neuroprotective interventions in term newborns is vital.^8^ Neuroimaging biomarkers have been used in clinical trials such as the Lactate/NAA ratio on spectroscopy on MRI^9^ but serial measurements are not readily available at multiple early timepoints or through childhood. Therefore, inflammatory markers have shown promise as prognostic biomarkers for outcomes of NE.^3^ Clinical trials are increasingly utilising blood biochemical biomarkers as surrogate outcomes rather than waiting several years for neurodevelopmental outcomes. These biomarkers may potentially help to stratify patient groups for suitable interventions in more complex study designs.

## High-mobility group box protein 1 (HMGB1) in NE

Rui et al. retrospectively analysed 216 infants both term and preterm diagnosed with encephalopathy and found those with a poor prognosis had increased serum high-mobility group box protein 1 (HMGB1).^10^ Decreases in HMGB1 over time were associated with a good prognosis but increases were associated with a poor prognosis. An Apgar score of 0–3 at 5 min, extremely preterm birth, premature rupture of membranes, moderate to severe NE and serum HMGB1 > 6.14 ng/mL were associated with a poor prognosis as an independent risk factor. HMGB1 levels correlated with Interleukin (IL)-6 and CRP indicating inflammatory status in NE. This paper included predominantly extremely low birthweight infants and only 40 term infants were included and details of therapy such as TH were not reported.^11–14^ The authors extrapolated term infant definitions of NE to preterm infants which is controversial as there are currently no consensus guidelines for encephalopathy in preterm infants.^15^ The American College of Obstetricians and Gynecologists (ACOG) describes NE as a “clinically defined syndrome of disturbed neurologic function in the earliest days of life in an infant born at or beyond 35 weeks of gestation, manifested by a subnormal level of consciousness or seizures, and often accompanied by difficulty with initiating and maintaining respiration and depression of tone and reflexes”.^10^ This heterogeneous patient group including term NE and preterm infants with HI highlight the need for further studies using consensus definitions and stratifying by gestation.

High mobility group box 1 protein (HMGB1) is also named amphoterin and high-mobility group protein 1 (HMG-1) and is encoded by the *HMGB1* gene.^16^ Nuclear HMGB1 inside the cell, regulates the structure and function of chromosomes and is bound extensively to DNA which is important in DNA replication, telomere maintenance, nucleosome assembly and transcriptional regulation. Extracellular HMGB1 can be passively released by necrotic tissue or stressed cells or actively secreted and mediates inflammation, metabolic responses and immunity. It acts as a cytokine and is a damage-associated molecular pattern (DAMP) protein binding to pattern recognition receptors (PRR)such as Toll-like receptors^17^ and receptor for advanced glycation end products (RAGE), which control proinflammatory cytokine release. Targeting HMGB1 release and activity has potential for the treatment of neonatal sepsis and encephalopathy.

## HMGB1 as a therapeutic target in NE

Therapeutic hypothermia remains the only standardised treatment with the decrease of core temperature to 33–34 °C for 72 h and a number needed to treat of 7. However ~50% of survivors have disability^18,19^ and adjunctive therapies are the focus of ongoing studies. HMGB1 has been implicated in neurological disorders including sepsis-induced encephalopathy in adults.^20^ Although there are limited data available about the role of HMGB1 in neuroinflammation following sepsis, it has been implicated in other neurologic disorders and causes blood-brain barrier damage when translocating to the extracellular space from the nucleus causing neuroinflammatory responses. Anti-HMGB1 antibodies and antagonists such glycyrrhizin or HMGB1 interference (shRNA)inhibit the neuroinflammatory response post-traumatic brain injury and subarachnoid haemorrhage (SAH).^21^

Neonatal microglia in hypoxic ischemic encephalopathy have upregulated HMGB1 and can alter M1/M2 phenotypic polarization, leading to cortical injury. HMGB1 could be a factor in HI-related brain injury in the newborn, analogous to adult stroke. HMGB1 was upregulated in activated microglia after HI in a neonatal model and inhibition of HMGB1 with glycyrrhizin decreased hippocampal neuronal and microglial loss and neurobehavioral abnormalities were reduced.^11,22^

## Future directions

Larger human neonatal studies or collaborative projects including term infants with neonatal encephalopathy detailing study design, aetiology and management would allow the validation of HMGB1 and correlation with short and longer-term outcomes. In vitro human immune response to glycyrrhizin could be developed for future human clinical trials. Therefore, HMGB1 may act as biomarker of injury and also a target for immunomodulation in clinical trials.
